# Multigene and Improved Anti-Collision RRT* Algorithms for Unmanned Aerial Vehicle Task Allocation and Route Planning in an Urban Air Mobility Scenario

**DOI:** 10.3390/biomimetics9030125

**Published:** 2024-02-21

**Authors:** Qiang Zhou, Houze Feng, Yueyang Liu

**Affiliations:** School of Electronic and Information Engineering, Beihang University, 37 XueYuan Road, Haidian District, Beijing 100191, China; sy2102512@buaa.edu.cn (H.F.); sy2202315@buaa.edu.cn (Y.L.)

**Keywords:** UAVs, UAM scenario, task allocation, path planning, multigene algorithm, RRT* algorithm

## Abstract

Compared to terrestrial transportation systems, the expansion of urban traffic into airspace can not only mitigate traffic congestion, but also foster establish eco-friendly transportation networks. Additionally, unmanned aerial vehicle (UAV) task allocation and trajectory planning are essential research topics for an Urban Air Mobility (UAM) scenario. However, heterogeneous tasks, temporary flight restriction zones, physical buildings, and environment prerequisites put forward challenges for the research. In this paper, multigene and improved anti-collision RRT* (IAC-RRT*) algorithms are proposed to address the challenge of task allocation and path planning problems in UAM scenarios by tailoring the chance of crossover and mutation. It is proved that multigene and IAC-RRT* algorithms can effectively minimize energy consumption and tasks’ completion duration of UAVs. Simulation results demonstrate that the strategy of this work surpasses traditional optimization algorithms, i.e., RRT algorithm and gene algorithm, in terms of numerical stability and convergence speed.

## 1. Introduction

Unmanned aerial vehicles (UAVs), also known as drones, have attracted lots of attention from the industry and academia, owing to their versatility, flexibility, and coordinated swarming capabilities [1]. UAVs are primarily categorized into two fixed-wing and rotary-wing UAVs [2]. Unlike fixed-wing UAVs, the latter can hover at fixed sites and vertically takeoff or land without runways or launchers. Therefore, the deployment and collaboration of rotary-wing UAVs offers a practical solution to alleviate congestion in ground transportation and delivery backlogs in urban environments [3]. In this context, Urban Air Mobility (UAM) scenario, demonstrated by Uber in [4], uses a type of rechargeable rotary-wing UAV called electric Verticals Take-Off and Landing (eVTOL) to balance the delivery of goods and passenger transport.

The industrial landscape of applying UAVs in the logistics and transportation sectors is also in progress. For instance, Walmart employed automated UAVs to deliver goods to customers in rural areas. DHL, one of Europe’s biggest mailing companies, tested a new automation UAV-based platform for providing services. Regarding aeronautical manufacturing enterprises, e.g., NASA, Uber, and Hyundai Motor have focused their attention on the architectural and technical challenges of integrating automated UAVs into UAM transportation systems [2]. A key technique challenge in UAM is the strategic deployment of UAVs for provision services. The procedures of UAV deployment can be mainly classified into route planning and task allocation [5].

In terms of task-allocation problems, collaboration among UAVs is crucial to reduce conflicts and enhance efficiency. Hao-Xiang et al. put forward a modified organism search algorithm to solve task assignment problems with multiple UAVs [6]. The work [7] proposed an adaptive genetic algorithm to assign multiple heterogeneous UAVs performing military tasks within a limited time. Randal et al. studied cooperative target assignments under enemy threats.

Mathematical models of previous works mainly include Mixed Integer Linear Programming (MILP), Multiple Traveling Salesman Programming (MTSP), Multiple Vehicle Routing Programming (MVRP) and Cooperative Multiple Task Assignment Programming (CMTAP) [8,9,10,11]. The MILP model is widely used in task assignment problems owing to its extensibility. However, the simple processes of MILP models are more suitable for small-scale problems. Additionally, the MTSP model does not consider the heterogeneity of tasks. Additionally, the MVRP model suits well time-constrained tasks, but does not refer to the flight dynamic performance of UAVs [12,13,14]. On the contrary, the CMTAP model is suitable for heterogeneous task assignments with multiple UAVs. Therefore, the CMTAP model of this work is built and studied for task assignment problems with multiple UAVs in a UAM scenario.

As for addressing the CMTAP model of task assignment problems, genetic algorithms are commonly used for their scalability, simplicity, and extensibility. However, the failure of a single UAV can cause deadlock in the genetic algorithm. Moreover, different task constraints for heterogeneous tasks can lead to the local existence of the solution. In this regard, the work improves and proposes the efficient and innovative multigene algorithm by designing customized selection and multipoint crossover for heterogeneous tasks. Specific details will be expanded in the following sections [15,16].

The main goal of the trajectory design of multiple UAVs is to improve energy efficiency and enhance the timeliness of the system. Huimin et al. optimized UAVs’ trajectory to minimize time-related performance [17]. They presented a novel framework to solve the realistic non-convex problem. The work [18] adopted the genetic algorithm and the particle swarm algorithm in optimizing the trajectories of UAVs. The simulation proves that the genetic algorithm performs better than the particle swarm algorithm [19].

According to route planning problems of multiple UAVs in previous works, an efficient path planning algorithm can improve the execution efficiency and robustness of the system. Therefore, reducing the obstacle avoidance system’s discovery time and path length is worthy of research. Hassan et al. proposed a novel hybrid form of the Dubins-simulated annealing (HDSA) optimization framework for emergency landing, which can plan the most suitable and admissible trajectory to the landing site in emergency flight conditions [20]. Dolgov et al. proposed the A* algorithm, a graph traversal algorithm, to find the optimal path in an obstacle avoidance system [21]. However, the computation load of each step increases fast with the growth of the complexity of the environment [22,23]. The rapidly exploring random tree (RRT) algorithm has the integrity of spatial search ability to fast and randomly sample in the map without pre-modeling [24]. However, the convergence speed of the RRT algorithm is low, and the smoothness of the generated path is poor due to the randomness of the search direction [25,26,27,28,29].

Previous works have improved and optimized the RRT algorithm to address its shortcomings in the efficiency of path planning. The RRT* algorithm proposed by Karaman et al. adopts two strategies, reselecting parent nodes and rewiring, to plan a near-optimal path, which may increase time cost [30]. Luo et al. proposed an informed RRT* algorithm based on Karaman’s work. The informed RRT* algorithm limits the sampling area to an ellipse to improve the speed of obtaining the asymptotic optimal path [31]. Kuffner et al. proposed the RRT Connect algorithm, which adds one more random tree with the target point as the root node [32,33]. Two random trees of the RRT Connect algorithm grow towards each other simultaneously [34].

Although scholars have performed a lot of previous work on improving the RRT* algorithm to solve route planning problems, there still exists poor adaptability to the UAM scenario, slow convergence speed in finding the target, and poor planning path quality [35,36]. To address the above shortcomings, this work proposes an improved anti-collision RRT* (IAC-RRT*) algorithm. First, the IAC-RRT* algorithm offers the map complexity evaluation strategy to calculate the corresponding map’s suitable step size and bias probability. The time switching factor is also used to adapt the UAM scenario. Moreover, the normal line between the source and the goal is introduced to ensure the random tree explores positively to the target point. Simulation results have demonstrated the IAC-RRT* algorithm can improve the effectiveness and quality of sampling points, leading to better planning path quality.

Overall, the main contributions of this paper are listed as follows:

- To the best of our knowledge, we first study to minimize energy consumption in the context of heterogeneous task assignments for multiple UAVs in urban environments. This research not only tackles the task assignment problem but also considers charging requirements and diverse task types. Consequently, the task assignment problem constitutes a CMTAP model. To address this model, we propose a multigene algorithm by designing customized selection and multipoint crossover mechanisms to handle the heterogeneity of tasks.
- This paper also proposes the IAC-RRT* algorithm to compute the route planning problems in the UAM scenario. A time-switching factor is proposed to adapt the airspace management regulations of the UAM scenario. Moreover, the normal line between the source and the goal is introduced to ensure the random tree explores positively to the target point. Compared to the RRT* algorithm, the novel route planning method works well in urban environments.
- Simulation results indicate that the strategy of this work exhibits superior adaptability and efficiency than the traditional optimization algorithm, i.e., RRT* algorithm, and gene algorithm. This advancement marks a significant stride in optimizing UAV schedules within UAM scenarios.

## 2. Models and Methods

### 2.1. System Model

**Figure 1:**
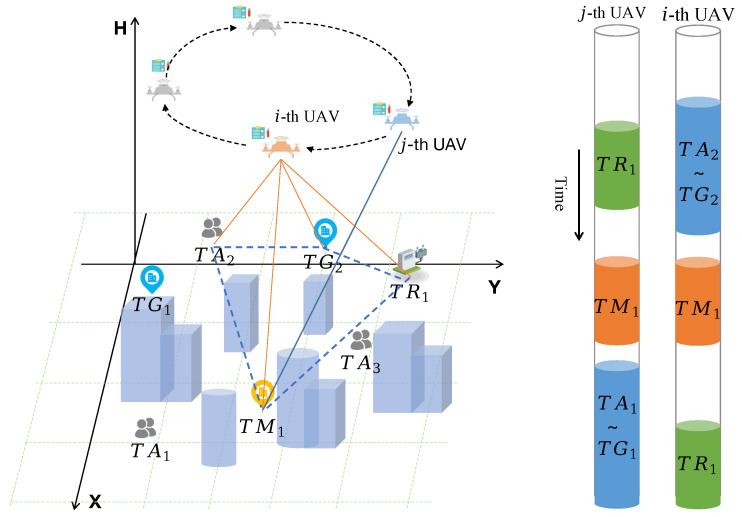
System model of UAV-enabled task scheduling system in UAM scenario.

We consider a UAV-enabled task scheduling system in UAM scenarios with UAVs, u∈U={1,2,…,Nu}, and tasks, s∈S={1,2,…,Ns}, as shown in Figure 1, where U and S are sets of UAVs and tasks, respectively. Additionally, the number of UAVs and tasks are denoted as Nu and Ns. Each UAV has communication and navigation devices, allowing persistent real-time communication with the ground control center. Heterogeneous tasks, randomly generated and distributed in urban space, are divided into different classes according to the traffic demand, which will be discussed in the following subsection. Nu UAVs cooperatively visit Ns known task sites in clusters, and the IAC-RRT* algorithm is employed to optimize the trajectory of each UAV, thereby guaranteeing the minimization of flight paths while adhering to safety protocols.

The location of the *s*-th task point is denoted by coordinates $\left(x_{s},y_{s},h_{s}\right)$, where s∈S. Similarly, the position of the *u*-th UAV at a given time *t* is denoted by $\left(x_{u}^{t},y_{u}^{t},h_{u}^{t}\right)$. Although tasks are heterogeneous, the mechanical energy consumption of UAVs during the task execution is not incorporated in the total energy consumption in subsequent problem formulations.

#### 2.1.1. Heterogeneous Task Execution Model

As for UAV tasks, the *k*-th task generated at time *t* is denoted as

(1) $$s_{l}^{t}\left(k\right)=\left\{F_{l}\left(k\right),D_{l}\left(k\right),\left(x\left(k\right),y\left(k\right),h\left(k\right)\right)\right\},$$

where l∈1,2,3 represents different task types, and $F_{l}\left(k\right)$ denotes the total number of UAVs are required to finish the *k*-th task, which is defined as

(2) $$F_{l}\left(k\right)=\left\{\begin{array}{cc} 1, & \mathrm{single}-\mathrm{UAV}\;\mathrm{mission},\\ \{n|\;n\geq 2,\;n\in N+\}, & \mathrm{multi}-\mathrm{UAVs}\;\mathrm{mission}, \end{array}\right.$$

where $D_{l}\left(k\right)$ represents the duration of completing the task, which remains consistent for tasks of identical type. And (x(k),y(k),h(k)) is the position of a specific task $s_{l}^{t}\left(k\right)$. In this paper, UAV missions are divided into three typical types, each tailored to meet the demands of the UAM scenario.

Air taxi task I: This task type is denoted by l=1. As shown in Figure 1, there are Na air taxi tasks in the UAM scenario, e.g., UAV *i* transports loads in demand from one designated site $TA_{2}$ to another $TG_{2}$. The subscripts of TA and TG are numbered sequentially. Additionally, this is the single-UAV mission type and $F_{l}=1$. The air taxi task is marked in blue from the timeline of the left part of Figure 1.

Hover and monitor task II: This task type is denoted by l=2. Multi-cooperated UAVs can be used as effective tools to hover at fixed locations and monitor some specific applications in UAM scenarios, i.e., precision agriculture, smart traffic monitor. To mathematically measure the problem of this type, we define a general task type with multi-cooperated UAVs performing tasks, and thus $F_{l}\geq 2$. Moreover, assigned UAVs cooperate and hover at task sites for a given length of time under unified control. Additionally, there are Nh hover and monitor tasks in the task set T.

Recharge task III: This task type is denoted by l=3, and $F_{l}=1$. Considering the battery capacity and the flight endurance of rotary-wing UAVs, charging stations are established in specific urban areas. Drones with low battery status can autonomously navigate to these stations for recharging. It is worth noting that each UAV’s battery capacity is defined as *C*, with three operational modes to ensure drone safety.

The battery level restricts task types that UAVs can accept. Firstly, the *i*-th UAV can not take air taxi task I if its current battery level $C_{i}\left(t\right)$ is less than 0.4C. Then, if the *i*-th UAV is in the middle of some task and its current battery level is less than 0.3C, it will flee back to the charge station once it finishes the task. Lastly, the *i*-th UAV is forced to fly back to the charge station when its battery level is less than 0.2C, regardless of whether it is in the middle of the task. However, if the UAV leaves its current task, this task will be judged as a failure. Therefore, task types taken by the *i*-th UAV with different battery levels are given as

(3) $$\begin{array}{c} \left\{\begin{array}{cc} s_{1}\left(k\right),\;s_{2}\left(k\right), & 0.4C\leq C_{i}\left(t\right)\leq C,\\ s_{2}\left(k\right), & 0.3C\leq C_{i}\left(t\right)\leq 0.4C,\\ s_{2}\left(k\right),\;s_{3}\left(k\right), & 0.2C\leq C_{i}\left(t\right)\leq 0.3C,\\ s_{3}\left(k\right) & C_{i}\left(t\right)\leq 0.2C. \end{array}\right. \end{array}$$

In (3), $s_{1}\left(k\right)$, $s_{2}\left(k\right)$, $s_{3}\left(k\right)$ are used to represent air taxi task I, hover and monitor task II and recharge task III.

#### 2.1.2. Energy Consumption Model

**Figure 2:**
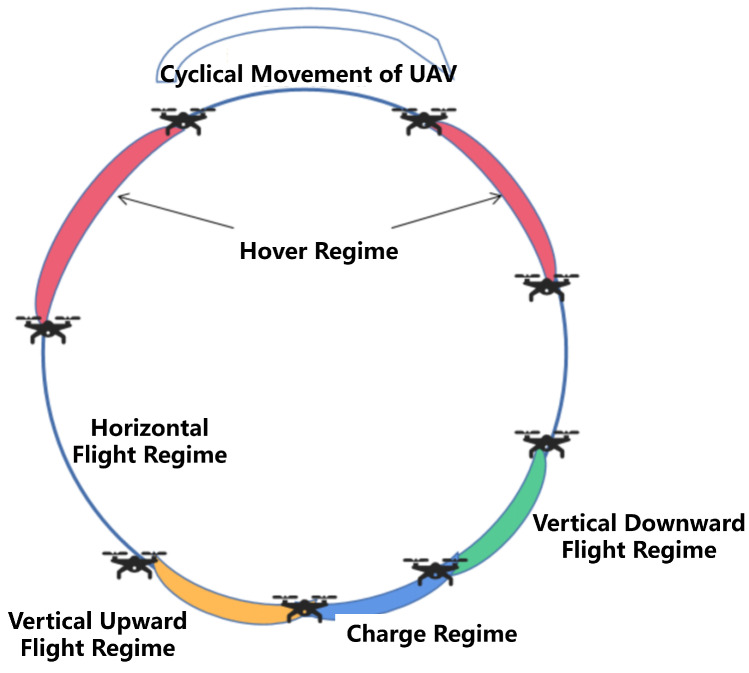
Cyclical movement of UAV.

This subsection illustrates the energy model for a UAV during a cruise. Within the specific scenario as shown in Figure 2, the analysis of energy consumption during UAV movements is divided into four distinct phases, contingent upon the UAV’s state of motion. We assume $G_{V}$ as the standard gravity of the UAV. The rotary-UAV’s thrust can be calculated as $F_{V}=\sqrt{(G_{V}-{\left(a_{1}{\left(V_{hor}\cos\left(\alpha \right)\right)}^{2}+a_{2}F_{V}\right)}^{2}+(a_{3}V_{hor}^{2}{)}^{2}}$. $V_{hor}$ and $V_{hor}$ are horizontal speed and vertical speed, respectively. α is the directional angle between the next target location and its orientation to the next waypoint. Although the flight dynamics of UAVs are intricate, any flight maneuver executed by a UAV can be decomposed into horizontal and vertical components. When calculating the energy consumption of complex flight movements, it can be effectively represented by summing the energy expenditures of both horizontal and vertical flight motions.

(1) UAV horizontally flying regime: In this state, UAVs horizontally fly during its transit. Suppose the horizontal flight velocity remains constant at $V_{t}$, and the consumption power $P_{I}\left(V_{t}\right)$ for horizontal flight is expressed as [37]

(4) $$\begin{array}{c} P_{I}\left(V_{t}\right)=\left(\frac{k_{1}}{k_{2}}+a_{4}\right){F_{V}}^{3/2}+a_{3}V_{t}^{3}. \end{array}$$

(2) UAV hovering regime: Similarly, the power consumption during UAV hovering $P_{H}$ is represented as [37]

(5) $$\begin{array}{cc} P_{H}= & =\left(\frac{k_{1}}{k_{2}}+a_{4}\right)G_{v}^{3/2}, \end{array}$$

where charging stations are located at ground level, necessitating UAVs to ascend or descend for recharging or task execution.

(3) UAV vertically flying upward regime: By neglecting the acceleration and deceleration process, UAVs ascend or descend at a constant velocity $V_{u}$. The ascending related power denoted by $P_{A}$ is calculated by [37]

(6) $$\begin{array}{c} P_{A}\left(V_{u}\right)=k_{1}G_{v}\left[\frac{V_{u}}{2}+\sqrt{{\left(\frac{V_{u}}{2}\right)}^{2}+\frac{G_{v}}{k_{2}^{2}}}\right]+a_{4}G_{v}^{3/2}. \end{array}$$

(4) UAV vertically flying downward regime: the descending related power denoted by $P_{D}$ with $V_{u}$ is formulated as [37]

(7) $$\begin{array}{c} P_{D}\left(V_{u}\right)=k_{1}G_{V}\left[\frac{-V_{u}}{2}+\sqrt{{\left(\frac{V_{u}}{2}\right)}^{2}+\frac{G_{v}}{k_{2}^{2}}}\right]+a_{4}G_{v}^{3/2}. \end{array}$$

In (4)–(7), $k_{1}$, $k_{2}$, $a_{1}$, $a_{2}$, $a_{3}$, and $a_{4}$ are technical parameters of vehicles, independent of the UAV’s motion or environment factors. Therefore, the energy consumption analysis for the *k*-th flight of UAV *i* can be segmented into four components corresponding to the movement states. The total energy consumption of UAV *i*’s *k*-th flight, $W_{i}^{k}$, can be given as

(8) $$W_{i}^{k}=\int_{0}^{\tau_{i}^{k}}P_{i}^{k}\left(t\right)\;dt=\underset{Horizontal\;flight}{\underset{︸}{\sum_{k=1}^{N_{j}+1}P_{I}\left(V_{t}\right)\frac{d_{j,i}^{k}}{V_{t}}}}+\underset{Hover}{\underset{︸}{\sum_{n=1}^{N_{j}}P_{H}\tau_{i,j}^{k,H}}}+\underset{Ascent}{\underset{︸}{P_{A}\left(V_{a}\right)\frac{H_{k}}{V_{a}}}}+\underset{Descent}{\underset{︸}{P_{D}\left(V_{d}\right)\frac{H_{k}}{V_{d}}}},$$

where $N_{j}$ is the total tasks that UAV *i* should finish in its *k*-th flight. $d_{j,i}^{k}$ is the distance between the site of task *j* and its previous site. $\tau_{i}^{k}$ is the *k*-th total flight duration of UAV *i*. $\tau_{i,j}^{k,H}$ is the hovering duration at site of task *j*. $H_{k}$ is the sum of elevated height in its *k*-th flight. Additionally, $V_{a}$ and $V_{d}$ are the velocities of upward and downward flight, respectively.

### 2.2. Problem Formulation

#### 2.2.1. Task Allocation Problems

The goal of task allocation is to achieve the optimal assignment of Ns tasks to Nu UAVs to maximize the overall reward. Additionally, the task list comprises Na air taxi tasks, Nh hover and monitor tasks, which satisfies

(9) Ns=Na+Nh,

and each task is exclusively assigned to a specific vehicle during a single flight. Furthermore, each UAV can be assigned a maximum of $L_{t}$ tasks, subject to the condition

(10) $$Ns<Nu\cdot L_{t}.$$

The global reward function is the summation of local rewards for each vehicle, which is determined by a function of the tasks assigned to each UAV. Optimization goals of time and energy consumption are studied separately. The time-oriented problem focuses on minimizing the time required to complete all tasks in an urban scenario, which can be mathematically represented as

(11) $$\begin{array}{cc} P_{1}:\; & \;\min\sum_{i=1}^{Nu}t_{i}\\ \; & s.t.\\ \; & \;t_{i}=\underset{flight\;time\;between\;task\;points}{\underset{︸}{\sum_{p}^{Ns}\sum_{j}^{Ns}S_{jp}^{i}\times t_{jp}^{i}+\sum_{p}^{Ns}S_{pa}^{i}\times t_{pa}^{i}}}+\underset{duration\;of\;task\;j}{\underset{︸}{\sum_{j=1}^{Ns}D_{j}^{i}\times t_{j}^{d}}}\\ \; & \;\;\;\;\;\;\;\;\;\;\;+\underset{charging\;duration}{\underset{︸}{\sum_{i}^{Nu}RE_{a}^{i}\times t^{c}}}\\ \; & \;D_{j}^{i}=\left\{\begin{array}{cc} 1, & \mathrm{if}\;UAV\;i\;handles\;task\;j,\\ 0, & \mathrm{otherwise} \end{array}\right.\\ \; & \;S_{ak}^{i}=\left\{\begin{array}{cc} 1, & \mathrm{if}\;UAV\;i\;handles\;tasks\;a,\;j,\\ 0, & \mathrm{otherwise}, \end{array}\right.\\ \; & \;RE_{a}^{i}=\left\{\begin{array}{cc} 1, & \mathrm{if}\;UAV\;i\;charges\;at\;site\;a,\\ 0, & \mathrm{otherwise} \end{array}\right. \end{array}$$

where $t_{j}^{d}$ is the operation time of task *j*, which is uniform for all UAVs. $t^{c}$ denotes the fixed charging duration, $t_{pa}^{i}$ signifies the flight duration between site *p* and *a*, and the charging station *a*. Constraints indicate that the operation time of a single UAV depends on whether the task is undertaken and which charging station is chosen.

The minimal power consumption problem is independent of the minimal time problem, as the power consumption of each vehicle determines the power consumption of completing all tasks in the system. According to Equations (4)–(8), the power consumption optimization problem of *k*-th flight can be represented by

(12) $$\begin{array}{cc} P_{2}:\; & \;\min\sum_{i=1}^{Nu}W_{i}^{k}\\ \; & s.t.\\ \; & \;i\in U,\;i\leq Nu,\\ \; & \;k\in N^{*}, \end{array}$$

where *k* belongs to the set of positive integers $N^{*}$, and represents any flight of UAV *i*.

#### 2.2.2. Route Planning Problems

The goal of route planning in this paper is to find the path with the shortest distance from the initial task site to the final task site within a limited time while avoiding obstacles in the UAM Operating Environment (UOE). The UOE, a flexible airspace area, can be redefined and modified over time. For example, if the traffic patterns at a nearby airport change, the available UOE may be adjusted to avoid potential traffic conflicts. Changes in the available UOE are likely to occur several times daily and can be promptly detected by the ground control center. Taking into account the dynamic nature of the UOE, the cost function for distance, $C_{length}$, can be represented by the node *i* [38],

(13) $$\begin{array}{cc} P_{3}:\; & \;\min\sum_{i=1}^{Nu}C_{length}\\ \; & s.t.\\ \; & \;D_{points}=\left\{i_{d}\;|i_{d}\in \mathrm{Discovered}\;\mathrm{Points}\right\},\\ \; & \;i_{d}\in Q_{n}+\gamma_{st}Q_{st},\\ \; & \;\gamma_{st}=\left\{\begin{array}{cc} 1, & \mathrm{if}\;\mathrm{normal}\;\mathrm{traffic}\;\mathrm{of}\;\mathrm{UAM}\;\mathrm{scenario},\\ 0, & \mathrm{otherwise}, \end{array}\right. \end{array}$$

where $\gamma_{st}$ represents the time transformation factor, which can be utilized to indicate the available UOE over time. The depreciation of (13) aims to identify the minimal $C_{length}$ encompassing the points $i_{d}$ discovered by the proposed algorithm within the available UOE. $Q_{n}$, $Q_{st}$, and $Q_{ob}$ are the general area, “flexible” area and obstacle area, respectively, where the “flexible” area can be controlled to switch by $\gamma_{st}$.

### 2.3. Proposed Algorithms

#### 2.3.1. Multigene Algorithm for Task Allocation Problem

**Figure 3:**
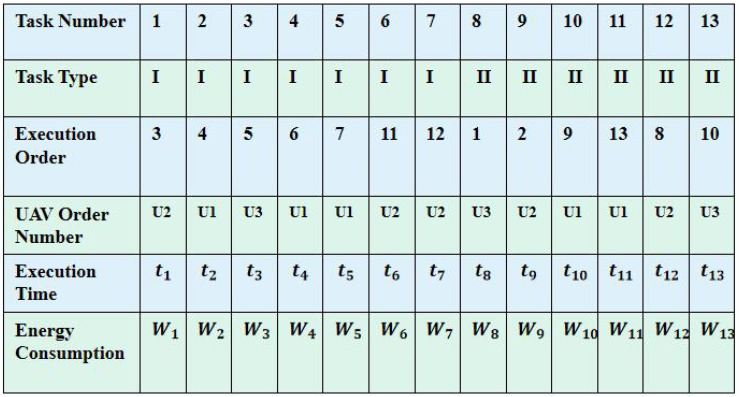
Figure of multigene chromosome coding method.

The initial step of the multigene algorithm involves the design of an encoding scheme that establishes connections between task sets, UAV sets, execution sequences, and assignment rules. Therefore, the proposed algorithm encodes the solution for (11) and (12) into a matrix form, also referred to as a chromosome, as shown in Figure 3. Each “gene” corresponds to a column with the matrix, representing a specific task assignment. In the first row of Figure 3, there are 10 tasks, comprising seven air taxi tasks labeled as 1–7, and three hover and monitor tasks labeled as 8–13. Additionally, No.8 and No.9 are associated with the same multi-vehicle task, necessitating the participation of two distinct vehicles. Rows two through six represent task type, execution order, UAV order number, execution time, and energy consumption.

The second step involves chromosome initialization, randomly generating $N_{chrom}$ encoding schemes. These $N_{chrom}$ initial chromosomes constitute the first generation of task allocation schemes. Subsequently, the initial set of chromosomes undergoes the iteration of crossover, mutation, and reinsertion until either the maximum number of iterations is reached or the optimal task allocation scheme is found. The processes mentioned above are outlined as follows.

**Figure 4:**
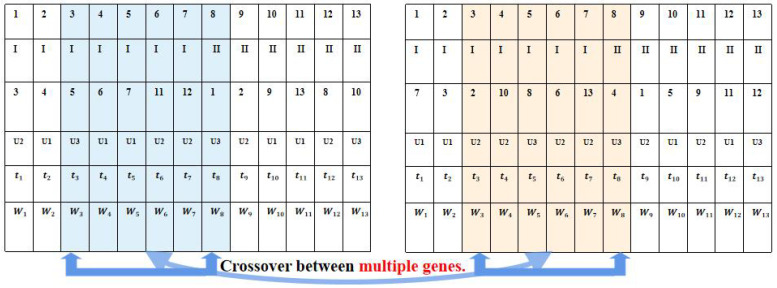
Figure of chromosome crossover.

Phase 1, crossover process: By employing roulette wheel selection, the selected chromosome randomly modifies segments of the gene sequence within its vicinity. The crossover probability $P_{c}$ varies depending on the task type and is defined as follows:

(14) $$\begin{array}{c} P_{c}=\left\{\begin{array}{cc} P_{c}\left(l=1\right), & l=1,\\ \frac{Na}{2Nh}P_{c}\left(l=1\right), & l=2, \end{array}\right. \end{array}$$

where $P_{c}\left(l=1\right)$ is set as a constant according to the simulation needs, Na and Nh represent the numbers of air taxi tasks and hover and monitor tasks, respectively. Given that the hover and monitor task requires two different UAVs, the crossover probability $P_{c}$ matching with the quantitative proportion of two task types can improve the search efficiency of the multigene algorithm. Furthermore, a partially matched crossover method ensures that each gene occurs only once, as shown in Figure 4.

**Figure 5:**
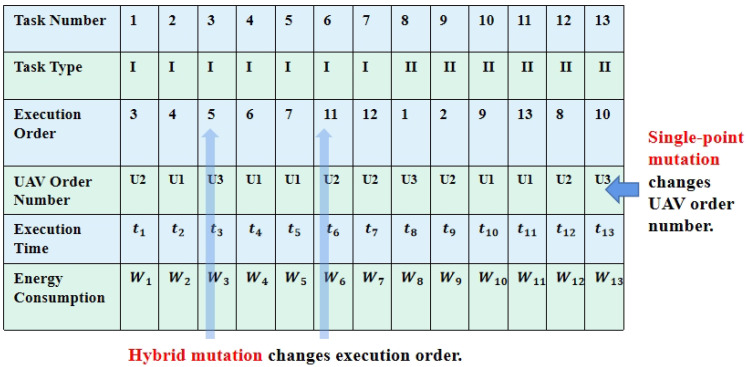
Figure of chromosome variation.

Phase 2, mutation process: In Figure 5, chromosome variation encompasses single-point mutation and hybrid mutation. The single-point mutation alters the UAV order number, while hybrid-point mutation modifies execution order. If the probability of single-point mutation and hybrid-point mutation set as a constant, $P_{m}$, the risk of losing excellent individuals increases. Therefore, the probabilities of single-point mutation and hybrid-points mutation are customized for each scheme based on its fitness value, *F*, in comparison to the average fitness value, $\bar{F}$, which are defined as

(15) $$\begin{array}{cc} F & =\frac{1}{\sum_{i=1}^{Nu}t_{i}},\\ \bar{F} & =\frac{1}{\sum_{i=1}^{N_{chrom}}F}. \end{array}$$

To accelerate the retention of superior schemes and the elimination of inferior schemes, the mutation probability of the current scheme is calculated as

(16) $$\begin{array}{c} P_{m}=\left\{\begin{array}{cc} \left(P_{m1}-P_{m2}\right)\cdot \ln\left(A\cdot F+B\right)+P_{m1}, & F\geq \bar{F},\\ P_{m1}, & F<\bar{F}, \end{array}\right. \end{array}$$

where $P_{m}$, $P_{m1}$, and $P_{m2}$ represent the current, minimum, and maximum values of mutation probability. *A* and *B* are functions designed to balance the relationship between mutation probability and fitness function, which are defined as

(17) $$A=\frac{e-1}{e(\bar{F}-F_{max})},$$

(18) $$B=\frac{\bar{F}-eF_{max}}{e(\bar{F}-F_{max})},$$

where *F*, $F_{max}$, and $\bar{F}$ are the fitness function’s current, maximum, and average values. *e* is Euler constant. Specifically, when calculating the fitness function value *F* for the current assignment, if $F\geq \bar{F}$, mutation probability should be set as the first case of (16). Otherwise, the mutation probability of the current scheme should be set to the maximum value of the mutation probability.

Phase 3, reinsertion process: The reinsertion process selects the chromosome with the best performance and inserts it into the next iteration. It ensures the subsequent generation maintains or improves upon the quality of the solution to the problem.

Overall, the process of the multigene algorithm has been organized and shown in Algorithm 1.
**Algorithm 1** Multigene algorithm for solving task allocation problem**Input:**      **U={1,2,…,Nu}, S={1,2,…,Ns}, Na, Nh;****Output:**      task allocation scheme with the highest *F*;1:Initialize $N_{chrom}$ schemes in initial chromosome;2:Calculate *F* of each scheme of the initial chromosome;3:Set $F_{max}$ and calculate $\bar{F}$ of the initial chromosome;4:$G_{e}$ = number of iterations;5:Set $P_{c}$, $P_{m}$, $P_{m1}$, $P_{m2}$;6:**while** $i\leq G_{e}$**do**7:   Crossover process in Phase 1;8:   Mutation Process in Phase 2;9:   Reinsertion Process in Phase 3;10:  Select the scheme with the highest *F* in this generation;11:  i=i+1;12:**end while**13:Output the scheme with the highest *F* in the last generation.

#### 2.3.2. Improved Anti-Collision RRT* (IAC-RRT*) Algorithm for Route Planning Problem

In this subsection, we proposed the IAC-RRT* algorithm, a robust and efficient algorithm for route planning designed to address the challenge of identifying feasible pathways within the UAM operation environment. The principles and concrete steps are as follows.

The first step involves map initialization and updating of task sites. The urban airspace area *Q* is divided into available area, $Q_{n}$, “flexible” area, $Q_{st}$, obstacle area, $Q_{ob}$ in Section 2.2.2. And the “flexible” area is controlled to switch by $\gamma_{st}$; the relationship is given as

(19) $$Q=Q_{n}+Q_{st}+Q_{ob},$$

then the start-point, $q_{start}$ and the end-point, $q_{end}$ are positioned within the domains $Q_{n}$ or $Q_{st}$. The task is deemed unsuccessful if these points fall outside the accessible regions, represented as

(20) $$q_{start},q_{end}\in Q_{n}\cup Q_{st}.$$

Subsequently, a direct line connecting $q_{start}$ and $q_{end}$ is established, designated as the normal line $R_{1}$, which aids in assessing exploration node efficiency.

The second step entails the construction of the tree structure. The algorithm explores the surrounding area with the radius $R_{e}$ from the root node $q_{start}$ and creates the newly found node $q_{new}$. The search and explore principle can be given that the smaller the included angle, $\theta_{ne}\left(i\right)$, between the normal direction and explore direction, the higher the chance of the node, $p_{ne}\left(i\right)$ to be chosen.

(21) $$p_{ne}\left(i\right)=\frac{1}{\pi /\theta_{ne}\left(i\right)}.$$

The algorithm determines whether the newly generated node $q_{new}$ belongs to $Q_{n}\cup Q_{st}$. If $q_{new}\in Q_{ob}$, the node will be discarded at once. Otherwise, the time transformation factor $\gamma_{st}$ will be used to determine whether the “feasible” area $Q_{st}$ would allow flight action.

Then, IAC-RRT* algorithm reselects neighbor node $q_{near}$ of $q_{new}$ within the radius of *R*, which is defined as

(22) $$q_{near}\in \left\{dis\left(q_{near},q_{new}\right)<R\right\},$$

By comparing the distance from $q_{near}$ to $q_{start}$ and $q_{new}$, the IAC-RRT* algorithm decides whether to change the parent node of $q_{new}$.

(23) $$\begin{array}{cc} \min\; & D_{new}=dis\left(q_{start},q_{near}\right)+dis\left(q_{near},q_{new}\right),\\ s.t.\; & q_{near}\in \left\{dis\left(q_{near},q_{new}\right)<R\right\}, \end{array}$$

the optimal reselected neighbor, denoted as $q_{near}^{*}$, becomes the new parent node of $q_{new}$. The rewiring process aims to reduce the path length across the global tree nodes, i.e., calculating the distance $D_{near}$ between each neighbor node and $q_{new}$, and selecting the minimal distance to $q_{start}$. The mathematical representation of the rewiring process is as follows:

(24) $$\begin{array}{cc} \min\; & D_{near}=dis\left(q_{start},q_{near}\right),\\ s.t.\; & q_{near}\in \left\{dis\left(q_{near},q_{new}\right)<R\right\},\\ \; & q_{new}\in \left\{parent\;nodes\;of\;q_{near}\right\}, \end{array}$$

These steps are reiterated until the distance between the newly generated node $q_{new}$ and $q_{end}$ is less than *R*. IAC-RRT* algorithm is described in Algorithm 2.
**Algorithm 2** IAC-RRT* algorithm for solving route planning problem**Input:**      $Q_{n}$,$Q_{st}$,$Q_{ob}$,$q_{start}$,$q_{end}$,*n* iteration times;**Output:**      Route from $q_{start}$ to $q_{end}$;1:Search from $q_{start}$;2:**while** Iterate *n* times **do**3:   **while** $dis(q_{new},q_{end})>R$ **do**4:     Generate $q_{new}$ within the radius *R*;5:     **if** $q_{new}\in Q_{ob}$ **do**6:        discard $q_{new}$;7:     **else** $q_{new}\in Q_{n}\cup \gamma_{st}Q_{st}$ **do**8:        reselect parent node for $q_{new}$;9:        $\min D_{new}=dis\left(q_{start},q_{near}\right)+dis\left(q_{near},q_{new}\right)$;10:       rewire with $q_{near}$ as the parent node;11:       $\min D_{near}=dis\left(q_{start},q_{near}\right)$;12:     **end if**13:   **end while**14:   Backtrack along $q_{new}$ from $q_{end}$ to $q_{start}$, i.e., find the route;15:   Select the route with minimal distance from each iteration;16:**end while**17:Output the selected route from $q_{start}$ to $q_{end}$.

## 3. Results

To evaluate the performance of the multigene algorithm and IAC-RRT* algorithm, we conduct simulations and compare the results with a few traditional task allocation and route planning algorithms. For all simulations without particular illustration, the parameters are fixed: we set the number of UAVs to 4, the number of tasks to 10, the number of air taxi tasks to 7, and the number of hover and monitor tasks to 3. The details of the simulations are presented as follows.

### 3.1. Simulation Results of Multigene Algorithm

In this subsection, we employ the gene and ant colony algorithms as comparative benchmarks. To assess the convergence probability, a series of experiments are conducted to search the most energy-efficient and quickest task allocation schemes.

**Figure 6:**
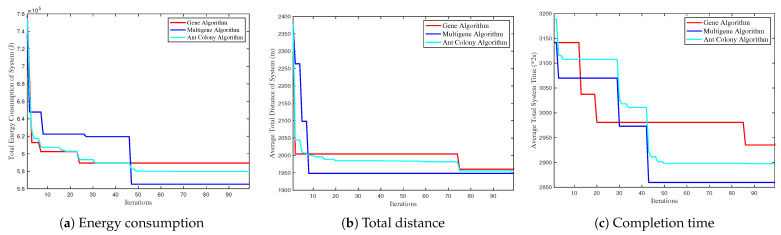
System performance of multigene compared with iterations. (**a**) Total energy consumption with iterations. (**b**) Average distance with iterations. (**c**) Average completion time with iterations.

Figure 6 limits the maximum iterations to 100 to ensure consistent experimental conditions, thereby evaluating the search performance of the multigene algorithm. Figure 6a–c demonstrate that our method significantly surpasses the gene and ant colony algorithms regarding search speed and optimal search outcomes, focusing on energy consumption, distance, and completion time. This superiority is attributed to the multigene algorithm’s targeted crossover and mutation, unlike the random approach of the other algorithms. Thus, we yield that heterogeneity-oriented crossover and mutation enhance the chance of discovering the optimal solution.

**Figure 7:**
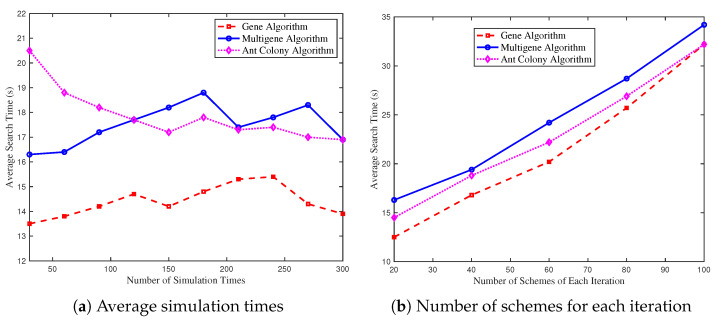
System performance comparison of three algorithms. (**a**) Average search time by varying average simulation times. (**b**) Average search time by varying number of schemes for each iteration.

Figure 7a depicts the relationship between the average system search time and the average number of simulation times. We can yield that with the increase in an average number of simulation times, the trend of average search time reveals no direct correlation. Additionally, the ant colony algorithm exhibits marginally inferior performance at lower simulation times. Figure 7b examines the association between average search time and the number of schemes of each iteration. A consistent trend is observed from Figure 7b. Given its superior search capabilities and outcomes, the extra search time invested in the multigene algorithm is deemed cost-effective.

**Figure 8:**
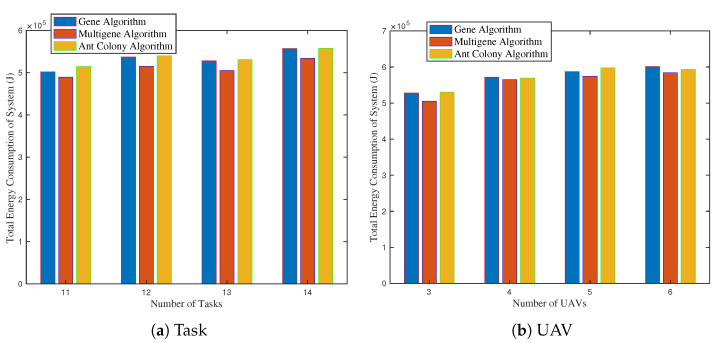
Energy consumption by varying number of tasks and UAVs. (**a**) Total energy consumption by varying number of tasks. (**b**) Total energy consumption by varying number of UAVs.

Figure 8a,b present the system’s total energy consumption by varying tasks and UAV numbers. As depicted in Figure 8a, the number of UAVs is set to 10. Additionally, we also set 11 tasks with 7 single-UAV tasks and 2 multi-UAV tasks, 12 tasks with 8 single-UAV tasks and 2 multi-UAV tasks, 13 tasks with 7 single-UAV tasks and 3 multi-UAV tasks, and 14 tasks with 8 single-UAV tasks and 3 multi-UAV tasks. We can conclude that total energy consumption increases with the number of tasks. Compared to the single-UAV tasks, the multi-UAV tasks will cause more significant growth in energy consumption. It can be explained by inherent complexity of heterogeneous tasks.

Figure 8b shows the system’s energy consumption variation by changing the number of UAVs. We can see that increasing the number of UAVs can not permanently reduce energy consumption. Notably, excessive UAVs can lead to inefficiencies and increased energy use. An inadequate number of UAVs can cause extreme round trips from charging stations to task sites.

**Figure 9:**
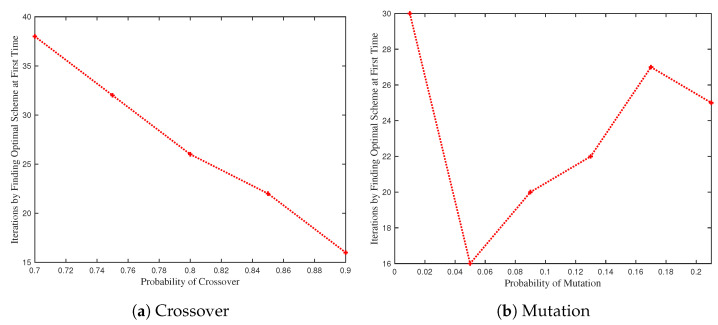
The number of iterations to find the optimal scheme by varying probabilities of crossover and mutation. (**a**) The number of iterations to find the optimal scheme by varying probability of crossover. (**b**) The number of iterations to find the optimal scheme by varying probability of mutation.

Figure 9 investigates the impact of crossover and mutation probabilities on the iterations required to identify the optimal scheme. We set schemes with system time under 6000 s as the optimal schemes. Figure 9a reveals an inverse relationship between crossover probability and required iterations, as a higher crossover probability enhances the chance of identifying more suitable task assignments. On the contrary, the iterations to find the optimal scheme vary with the likelihood of mutation Figure 9b. An increase in mutation probability risks losing excellent schemes, and an excessively low mutation probability may fail to generate sufficient options for optimal selection.

**Figure 10:**
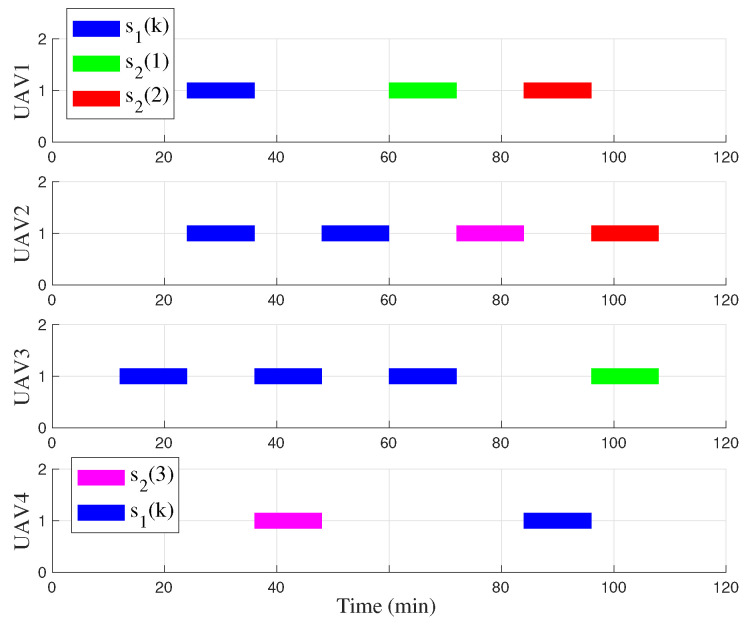
Task time schedule of four UAVs.

Figure 10 displays the UAV task schedule for an optimal task assignment. There are seven single-UAV tasks $s_{1}\left(k\right)$ and three multi-UAV tasks, color-coded for differentiation, $s_{2}\left(1\right)$, $s_{2}\left(2\right)$, $s_{2}\left(3\right)$. It can be observed that the optimal scheme balances fairness and effectiveness. Each UAV is assigned a similar number of tasks. However, the UAV to finish particularly challenging or isolated tasks, i.e., UAV4, may undertake fewer additional tasks.

### 3.2. Simulation Results of IAC-RRT* Algorithm

This subsection explores the performance of the IAC-RRT* algorithm. We simulated 4 UAV-assisted route planning scenarios in an urban environment. The environmental map is modeled as a $2000\times 2000\times 100\;m^{3}$ cube, which is divided into available areas, $Q_{n}$, “flexible” areas, $Q_{st}$, obstacle areas, $Q_{ob}$. Flexible areas $Q_{st}$, modeled as green box regions, are controlled by a time-switching factor $\gamma_{st}$. Obstacles $Q_{ob}$ are modeled as randomly located hemispherical regions. All UAVs start and end at (0,0,5). Task sites which have seven single-UAV tasks and three multi-UAV tasks are randomly distributed in $Q_{n}$. The radius of exploration sets as 5m. The RRT algorithm serves as a benchmark for comparison.

**Figure 11:**
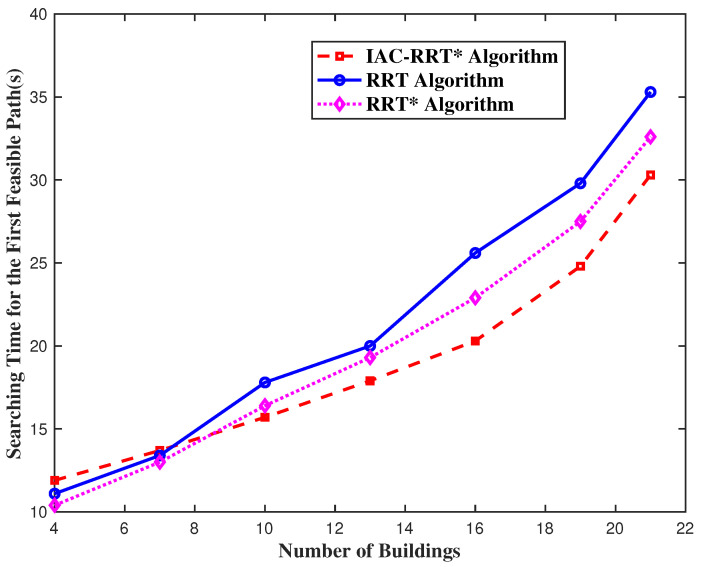
Searching time performance over different number of buildings.

**Figure 12:**
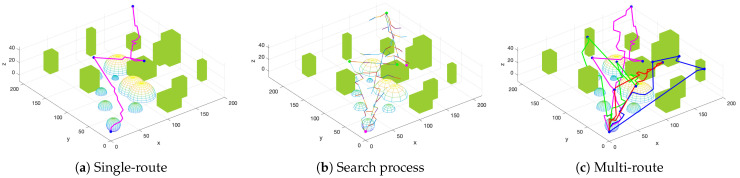
Routing planning map of IAC-RRT* algorithm in urban environment. (**a**) Single route found by IAC-RRT* algorithm. (**b**) Search process of IAC-RRT* algorithm. (**c**) Final planning route for all vehicles of IAC-RRT* algorithm.

Figure 11 represents the searching time performance of three algorithms over different building densities. To test the obstacle avoidance performance of the IAC-RRT* algorithm, RRT and RRT* algorithms work as comparison groups [25]. Accordingly, the gap is not distinct at low building density because random search methods may not easily bump into obstacles. However, searching methods along the normal direction have better effects at high building densities. Additionally, simulation results prove that the IAC-RRT* algorithm performs better than the RRT algorithm and RRT* algorithm in a complex obstacle environment. Figure 12 portrays the IAC-RRT* algorithm’s route search process. Figure 12a depicts the initial single route of the IAC-RRT* algorithm by finishing three tasks. Consequently, Figure 12b defines the search process of the IAC-RRT* algorithm. It can be obtained that exploration detection of the search process prioritizes the normal line R1 to increase the target discovery chance. Figure 12c depicts the final planned route for all vehicles of the IAC-RRT* algorithm. We can affirm the IAC-RRT* algorithm’s proficiency in task completion.

**Figure 13:**
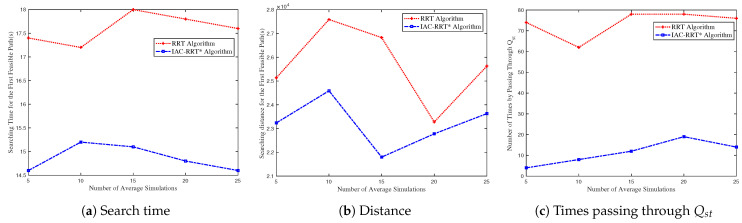
System performance of IAC-RRT* algorithm compared with RRT algorithm. (**a**) Searching time performance of IAC-RRT* algorithm compared with RRT algorithm. (**b**) The optimal feasible distance of IAC-RRT* algorithm compared with RRT algorithm. (**c**) Times passing through $Q_{st}$ of IAC-RRT* algorithm compared with RRT algorithm.

Figure 13 compares the system performance of the IAC-RRT* algorithm and RRT algorithm. Figure 13a shows the IAC-RRT* algorithm’s faster convergence time by 13% compared to the RRT algorithm. Then, Figure 13b depicts the search distance performance of the IAC-RRT* algorithm. The variation in the number of average simulations between 5 and 15 owes to too few experiments. It can also be obtained that converge speed of the IAC-RRT* algorithm is quicker 12% than the RRT algorithm. Figure 13c demonstrates times passing through $Q_{st}$ of the IAC-RRT* algorithm and RRT algorithm. The time-switching factor $\gamma_{st}$ reduces 85% per cent passengers through $Q_{st}$. Remaining points occurs in $Q_{st}$ of IAC-RRT* algorithm when $\gamma_{st}=1$.

## 4. Conclusions

In this paper, we propose the multigene algorithm and IAC-RRT* algorithm to solve UAV task allocation and route planning problems in the UAM scenario. Also, we studied the UAV power model and task model, and derived expressions of task allocation problems. Simulation results indicate that our algorithms have better numerical stability, which results in better solutions to the problem. The convergence speed of the proposed multigene algorithm is 20 percent faster than the traditional gene algorithm. Additionally, the IAC-RRT* algorithm can handle airspace control issues with robust performance. It can converge quicker 12% than the RRT algorithm.

## Data Availability

The raw data supporting the conclusions of this article will be made available by the authors on request.
